# Risk Profiles for Falls among Older Adults: New Directions for Prevention

**DOI:** 10.3389/fpubh.2017.00142

**Published:** 2017-08-02

**Authors:** William A. Satariano, Constance Wang, Melissa E. Kealey, Elaine Kurtovich, Elizabeth A. Phelan

**Affiliations:** ^1^School of Public Health, University of California Berkeley, Berkeley, CA, United States; ^2^Department of Medicine, Division of Gerontology and Geriatric Medicine, School of Public Health, University of Washington, Seattle, WA, United States; ^3^Department of Health Services, School of Public Health, University of Washington, Seattle, WA, United States

**Keywords:** aged, falls, neighborhood, walking, community, prevention, descriptive analysis

## Abstract

**Objective:**

To address whether neighborhood factors, together with older adults’ levels of health and functioning, suggest new combinations of risk factors for falls and new directions for prevention. To explore the utility of Grade-of-Membership (GoM) analysis to conduct this descriptive analysis.

**Method:**

This is a cross-sectional, descriptive study of 884 people aged ≥65 years from Alameda County, CA, Cook County, IL, Allegheny County, PA, and Wake and Durham counties, NC. Interviews focused on neighborhood characteristics, physical and cognitive function, walking, and falls and injuries. Four risk profiles (higher order interactions of individual and neighborhood factors) were derived from GoM analysis.

**Results:**

Profiles 1 and 2 reflect previous results showing that frail older adults are likely to fall indoors (Profile 1); healthy older adults are likely to fall outdoors (Profile 2). Profile 3 identifies the falls risk for older with mild cognitive impairment living in moderately walkable neighborhoods. Profile 4 identifies the risk found for healthy older adults living in neighborhoods with low walkability.

**Discussion:**

Neighborhood walkability, in combination with levels of health and functioning, is associated with both indoor and outdoor falls. Descriptive results suggest possible research hypotheses and new directions for prevention, based on individual and neighborhood factors.

## Introduction

Falls are the leading cause of fatal and non-fatal injuries among adults aged 65 years and older (older adults). Every year, approximately 30% of U.S. adults aged 65 years and older fall (1). Ten percent of those falls result in hospitalization or death. During 2014, approximately 27,000 older adults died because of fall; 2.8 million were treated in emergency departments for fall-related injuries, and approximately 800,000 of these patents were subsequently hospitalized. Direct medical costs related to falls in the U.S. were approximately $30 billion in 2010, and these costs are expected to increase to as much as $54.9 billion by 2020, according to data from the Centers for Disease Control and Prevention [https://www.cdc.gov/HomeandRecreationalSafety/Falls/fallcost.html; Bergen et al. (2)].

Given its clinical and public health significance, there is a need to understand the full scope or “heterogeneity” of falls, i.e., variation in the characteristics of those who fall as well as the location and circumstances of the fall itself (3, 4). Most research to date has focused on indoor falls (4–6). Less attention has been given to falls that occur outdoors, which represent 40–60% of all falls by some accounts (3, 4). Given that those who fall outdoors tend to be healthier and more fit than those who fall indoors, outdoor environmental hazards are thought to be implicated, with sidewalks and parking garages being identified as common sites of outdoor falls (3, 4, 7).

While this research, especially the introduction of new neighborhood variables, should expand our understanding of the heterogeneity of falls, it will be challenging. The current approach is to look for risk factors one at a time in saturated regression models adjusted for confounders and other relevant covariates. This approach, while useful for isolating the effect of known factors, does not provide much information on factors not previously examined nor does it provide a straightforward way to look for joint effects of variables across disparate domains. It may be useful, therefore, to look at the effects of individual and environmental factors that tend to occur together in profiles (e.g., routine exercise, history of depression, and residence in a walkable neighborhood) in relation to the occurrence of indoor and outdoor falls.

This exploratory descriptive analysis, then, is designed to determine whether an expanded set of neighborhood variables, together with standard measures of health and functioning, and an innovative analytic strategy, show promise for more detailed study of the heterogeneity of falls (5, 6). This, in turn, may suggest new combination of variables to be tested (i.e., hypothesis testing), which is beyond the scope of the current paper.

## Materials and Methods

### Sample

This report is based on the Healthy Aging Research Network Walking Study, a cross-sectional study of the association between functional capacity, the neighborhood environment, and walking of older adults living in four regions across the United States (8). The sample consists of 884 people aged ≥65 years identified through senior organizations in Alameda County, CA, Cook County, IL, Allegheny County, PA, and Wake and Durham counties, NC. See the study by Satariano et al. (8) for a detailed description of the sampling design.

The study protocol was approved by the Institutional Review Board at each of the participating universities: the University of California, Berkeley; the University of Illinois, Chicago; the University of Pittsburgh; and the University of North Carolina, Chapel Hill. The interviews were conducted between September 2005 and November 2007. Each respondent provided written, informed consent prior to completing the interview. See Table 1 for a description of the sample characteristics.

**Table 1:** Demographics and falls by site compared to county census data.

	Alameda County, CA		Allegheny County, PA		Cook County, IL		Wake and Durham Counties, NC		Total	
	Study (%)	County^a^ (%)	Study (%)	County (%)	Study (%)	County (%)	Study (%)	County (%)	Study (%)	*p* Value^b^
**Demographic variables**										
**Age (*n* = 884)**										
65–74	49.2	51.6	46.3	49.3	52.2	52.4	56.0	55.0	51.0	0.92
75+	50.8	48.4	53.7	50.7	47.8	47.6	44.0	45.0	49.0	
**Sex (*n* = 884)**										
Female	77.4	59.3	78.6	61.0	71.4	60.5	78.4	60.7	76.6	<0.0001
Male	22.6	40.7	21.4	39.0	28.6	39.5	21.6	39.3	23.4	
**Race (*n* = 866)**										
Other race	5.0	3.2	0.0	0.2	1.0	2.3	0.0	0.4	1.6	<0.0001
Two or more races	4.2	2.5	0.0	0.4	2.5	1.3	0.4	0.6	1.8	
African-American	15.8	14.2	22.5	7.8	21.0	20.3	35.7	20.5	23.8	
Asian	13.8	17.7	1.5	0.3	0.5	3.0	7.5	1.3	6.2	
White	61.3	62.4	76.0	91.3	75.0	73.2	56.4	77.2	66.6	
**Latino or Hispanic (*n* = 871)**								
Yes	5.8	8.6	0.5	0.4	3.0	5.8	1.7	0.8	2.9	0.10
No	94.2	91.4	99.5	99.6	97.0	94.2	98.3	99.2	97.1	
**Years of schooling (*n* = 872)**
0–11 years	7.9	NA^c^	18.9	NA	5.5	NA	15.7	NA	11.9	
12 years	19.2	NA	51.2	NA	32.8	NA	30.0	NA	32.6	
Over 12 years	72.9	NA	29.9	NA	61.7	NA	54.3	NA	55.5	
**Income (*n* = 680)**										
Less than $15,000	20.9	23.0	36.1	28.1	15.1	25.3	30.8	22.8	26.0	<0.0001
$15,000–$24,999	19.4	16.4	33.5	23.3	23.8	18.6	21.1	15.9	24.0	
$25,000–$49,999	32.7	27.3	23.4	28.9	38.9	28.5	25.9	28.9	29.9	
$50,000 or more	27.0	33.3	7.0	19.7	22.2	27.6	22.2	32.4	20.1	
**Fallen in the past 6 months (*n* = 878)**
No	75.8	NA^c^	87.1	NA	74.7	NA	87.4	NA	81.2	
Yes	24.2	NA	12.9	NA	25.3	NA	12.6	NA	18.8	
**Location of most recent fall, if any (*n* = 164)**
Indoors	36.7	NA^c^	57.7	NA	53.1	NA	58.6	NA	48.8	
Outdoors	63.3	NA	42.3	NA	44.9	NA	41.4	NA	50.6	
Do not know	0	NA	0	NA	2.0	NA	0	NA	0.6	

*^a^US Census 2000 data for adults 65+*.

*^b^Overall p-value for the chi-square statistic comparing study populations to county populations as measured by the 2000 Census across the four geographic sites*.

*^c^NA, Census data categorized in the same way restricted to adults aged 65+ not available*.

### Baseline Interview

The interview included both a questionnaire and direct assessments of physical performance. The questionnaire included demographic and socioeconomic factors; history of falls and injuries; physical function and activities of everyday life (9–11); cognitive function (12–15); depression (16); symptoms associated with walking difficulties; self-reported assessments of neighborhood characteristics (17); and levels of walking and other forms of physical activity. Direct measures of performance were also included, based on measures of walking speed, balance, and lower body strength (8, 18–20), and summarized as a modified version of the Short Physical Performance Battery (SPPB) to assess lower body function. The measure of 400 m walk was chosen as it represents a typical walking distance covered by an older person (21). See the study by Satariano et al. (8) for a more complete description of the modified SPPB.

### Measures

The purpose of the study is to ultimately understand where falls occur as well as the personal and neighborhood attributes of the person falling. Respondents were asked whether they had fallen in the previous 6 months, and whether the most recent fall occurred outdoors or indoors. This report reflects the location of the respondent’s most recent fall within the past 6 months. If the respondent fell more than once in the previous 6 months and the location of the most recent fall was different from the location of the previous fall, only information about the most recent fall would be recorded.

### Neighborhood Environment: Self-report

Measurement of the neighborhood environment was based in part on questions from an abbreviated version of the Neighborhood Environment Walkability Scale (NEWS) (17, 22). Fourteen variables were created from the NEWS questions examining primary type of buildings, primary type of housing, walking time to destinations, land-use mix/access to services, street connectivity, walking/cycling facilities, esthetics, pedestrian traffic safety, crime and safety, neighborhood satisfaction/social capital, parking, cul-de-sacs, hilliness, and barriers to walking (e.g., freeways, railway lines, and rivers).

### Neighborhood Environment: Geographic Information Systems

Three geographic information system (GIS) variables were included in the analysis (number of selected types of businesses within a radial distance of each participant’s residence, median block length for census tract of residence, and housing density for census tract of residence).

The GIS-derived neighborhood business density variable was based on geocoded environmental data within a 400-m buffer (radial distance) of each participant’s residential address (23). ESRI Business Analyst was used, which contains data from InfoUSA for businesses listed on January 1, 2006. Businesses that were possible walking destinations were categorized according to North American Industry Classification System codes and summed to create a count of the number of retail businesses within the 400 m buffer.

Street connectivity (e.g., median block length) and housing unit density were determined by the census tract of each participant’s residence. The U.S. Census 2000 data from the SF3 files was used to measure housing unit density. Median block length data from 2000 from the RAND Center for Population Health and Health Disparities was used to measure street connectivity.

### Other Study Variables

In addition to the full set of demographic, socioeconomic, health, and functional data noted previously, the analysis included study site, access to an automobile, and the number of years at the current residence. See Appendix A in Supplementary Material for a complete list of variables included in the analysis.

### Analytic Strategy

Grade-of-Membership (GoM) analysis was used to identify risk profiles (24–27). GoM is a special case of latent class/latent trajectories models, which uses an algorithmic approach to analyze complex data (28, 29). Sets of higher order interactions of independent variables that occur together are identified and are referred to here as “profiles.”

Grade-of-Membership analysis is a descriptive approach. GoM is a class of latent structure models. Whereas latent class analysis, another common latent structure model, is used for discrete mixtures, GoM is developed for continuous mixtures.

Grade-of-Membership is designed to identify higher-order interactions (Profiles 1, 2, 3, and 4) associated with outdoor and indoor falls. The profiles suggest hypotheses, which would be test separately, e.g., the independent effect of cognitive function through conditional regression analysis. Testing specific hypotheses, suggested by GoM, is extremely important, but beyond the scope of this introductory, descriptive paper.

Grade-of-Membership analysis was conducted with GoM3 software package following a standardized procedure (30). For this exploratory study, we chose to fit four profiles, rather than to screen and search for the optimal number of profiles that fit the data. Each individual has a matrix of four scores that sum to 1.0; each score reflects the extent to which the individual fits with each of the four identified profiles. Each individual is assigned to only one profile as the best fit for his or her combination of responses across the full range of individual and environmental variables. We used the cut point of GoM score >0.5 for a given profile to classify each individual into only one profile. Those with a score less than or equal to 0.5 were included in the mixed profile. Analyses were conducted between March and May 2015.

## Results

### Sample

Table 1 compares the distribution of key sociodemographic variables by study site between participants and the general population of residents aged ≥65 years in the corresponding county. These results are generally consistent with a 1984 national survey of senior center users. Overall, 18.8% (165/878) of the participants reported a fall in the previous 6 months (Table 1). Of the 163 who reported the general location of their most recent fall (two of the 165 respondents did not do so), 49.1% (80/163) indicated that the fall occurred indoors and 50.9% occurred outdoors (83/163). As noted previously, the percentage difference in the prevalence of indoor and outdoor falls is generally consistent with what is reported in the literature (4, 6, 31).

### Profiles

Four distinct profiles were identified, based on the data from the full sample of 878 respondents (Appendix A in Supplementary Material; Table 2). For ease of presentation, Appendix A in Supplementary Material reports the distribution of each of 62 variables (rows) by each of the four profiles (columns). From left to right, the columns report the variable name, response categories, and frequency distribution. Following convention, if the frequency for a variable category is 1.8 times its probability for being in the profile, then the variable category is considered to be a “distinguishing” feature of that profile. Each of the distinguishing variable categories is bolded for each of the four profiles (columns 5–8). Table 2 highlights the main points from Appendix A in Supplementary Material by listing the distinguishing variable categories for each profile.

**Table 2:** A summary of the distinguishing categories for the individual and environmental variables by profile.

Profile 1	Profile 2	Profile 3	Profile 4^d^
Frail older adults/poor neighborhood walkability	Healthy older adults/good neighborhood walkability	Cognitively impaired older adults/moderate neighborhood walkability	Healthy older adults/poor neighborhood walkability
Overall health fair/poor Health compared to others same Health worse compared to others Eyesight worse compared to others Hearing better compared to others Limit or avoid activities because of concern about falling Difficulty with >5 functional tasks ADL^a^ assistance needed 1 task ADL^a^ assistance needed >1 tasks Difficulty walking due to leg weakness Difficulty walking due to need to use bathroom Difficulty walking due to fatigue Difficulty walking due to problem with start/stop Difficulty walking due to chest pain Difficulty walking due to shortness breath Worry about fall some Worry about falls a lot MMSE^d^ lowest quartile Depressed^f^ Confidence walking 10 blocks low IADL^g^ limitations 1 IADL^g^ limitations 2 IADL^g^ limitations all 3 NEWS^b^ walk time to destinations longest quartile NEWS^b^ quality of walking places poor NEWS^b^ surrounding not attractive NEWS^b^ crime safety unsafe No access to vehicle Financial needs not adequately met Income <$15,000 Education 0–11 years African-American Housing density^c^ more dense quartile Count of businesses^c^ somewhat few Count of businesses^c^ more Lower body function^e^ lowest Years at address >50–60 years	Overall health excellent Male Health better compared to others No difficulty with functional tasks ADL^a^ no assistance needed No leg weakness No neck pain No problem with concentration No problem seeing steps No dizziness No problem with needing bathroom No fatigue NEWS^b^ surroundings somewhat attractive NEWS^b^ traffic safety excellent NEWS^b^ neighborhood satisfaction somewhat do know each other Access to vehicle Financial needs met very adequately Income $25,000–49,999 Currently employed Serves as caregiver Median block length short^c^ Lower body function^e^ somewhat high Years at address >50–60 years Number of close friends and/or relatives 11–100	Does not limit or avoid activities because of concern about falling ADL^a^ no assistance needed No leg weakness No problem standing tall No neck pain No problem with memory No problem seeing steps No problem with glare No dizziness No problem with balance No problem with needing bathroom No fatigue NEWS^b^ walk to services somewhat low accessibility NEWS^b^ quality of walking places somewhat poor NEWS^b^ traffic safety poor NEWS^b^ crime safety unsafe NEWS^b^ crime safety somewhat safe NEWS^b^ neighborhood satisfaction do not know each other NEWS^b^ difficulty parking No access to vehicle Financial needs met somewhat adequately Income <$15,000 Currently not employed Education 0–11 years Education 12 years African-American Other 1 race Other 2+ races Housing density^c^ most dense quartile Median block length^c^ shortest Count of businesses^c^ more Number of close friends and/or relatives 0–3	Does not limit or avoid activities because of concern about falling ADL^a^ no assistance needed No leg weakness No problem standing tall No problem with memory No problem with concentration No problem seeing steps No problem with glare No dizziness No problem with balance No problem with needing bathroom NEWS^b^ quality of walking places poor NEWS^b^ traffic safety excellent NEWS^b^ crime safety safe NEWS^b^ easy parking NEWS^b^ lots of cul-de-sacs Access to vehicle Income $50,000+ Asian Housing density^c^ least dense quartile Median block length^c^ longest Count of businesses^c^ few

*^a^Activities of Daily Living*.

*^b^Neighborhood Environment Walkability Scale*.

*^c^Geographic Information System variable*.

*^d^Modified Mini-Mental State Exam*.

*^e^Summary of direct measures of balance, walking speed, and lower body strength*.

*^f^Per Center for Epidemiologic Studies Depression Scale*.

*^g^Instrumental Activities of Daily Living*.

As noted previously, this is an exploratory analysis. The four profiles were employed as a “proof of concept,” an exploratory analysis to look at the usefulness of this type of model/results for hypothesis generation. It is not intended to a comprehensive identification of all possible profiles. We did not optimize the modeling or look at overall fit. We chose a 4-profile solution *a priori* based on prior experience with GoM analysis to see what results it would offer.

Again, this analysis is not designed to assess the independent effects of specific variables. Instead, we were interested in a descriptive analysis to assess the potential utility of identifying higher order interactions, which, in turn, suggest hypotheses for subsequent analyses.

Each of the four profiles is described below. A name was assigned to each profile based on its distinguishing individual and neighborhood features. The four profiles accounted for 75.4% (669/878) of the respondents. In contrast, 209 or 23.8% could not be so classified and were included in the mixed group.

#### Profile 1: Frail Older Adults/Poor Neighborhood Walkability

Of the 878 respondents, 13.3% (117/878) are best classified in Profile 1. Those in this profile are characterized by poor self-reported health; a variety of limiting symptoms; poor vision; depressive symptoms; poor self-reported cognitive function; and reduced cognitive performance, as measured by the Modified Mini-Mental State Exam. In addition to poor health and functioning, this profile is characterized by participants’ reports of the neighborhood being unsafe, with relatively long distances to important destinations, and unattractive surroundings. Finally, those in this profile are likely to be African-American with less than a high school education and an annual income of less than $15,000.

#### Profile 2: Healthy Older Adults/Good Neighborhood Walkability

Profile 2 includes 18.9% (166/878) of the participants. In contrast to Profile 1, the people in this profile are likely to both report and display very good health and functioning. In addition to excellent overall health, there are no reports of limitations associated with specific symptoms, such as leg weakness and shortness of breath. There are no reports of difficulties with ADLs, generic functional tasks, or cognitive function. Indeed, Profile 2 is associated with relatively high scores for both the objective cognitive texts as well as direct measures of physical performance in the highest quartiles. In addition to positive health and functioning, there are reports that are consistent with a very walkable neighborhood, including relatively short walking distance to important destinations. In addition to walkability, this profile is also characterized by access to other forms of mobility, such as driving. With regard to social factors, this profile is characterized by long-term residence of 50–60 years, living with a spouse, providing care to someone outside the home, a relatively large friendship network, being currently employed, with an annual income of $25,000–$48,999, and reports that their financial resources are adequate to meet daily needs.

#### Profile 3: Cognitively Impaired Older Adults/Moderate Neighborhood Walkability

Of the 878 respondents, 19.2% of them are best classified in Profile 3. In general, Profile 3 is characterized by respondents in reasonably good physical health and functional status. Although people in Profile 3 do not tend to report difficulties with cognitive function, they are likely to score in the lowest quartile of the objective cognitive measures. There are also mixed results for neighborhood walkability. While people in Profile 3 are likely to live in areas of high walkability, based on GIS data, they are likely to report general impediments to walking in their neighborhoods, including somewhat low confidence in being able to walk 10 blocks in their neighborhoods. It is also reported that driving and parking a car is difficult in their neighborhood. In terms of socioeconomic status, Profile 3 is characterized by participants with an annual income of less than $15,000, an education of less than 12 years, and being unemployed. Like Profile 1, African-American or being of mixed racial heritage is a distinguishing feature of Profile 3.

#### Profile 4: Healthy Older Adults/Poor Neighborhood Walkability

Nearly a quarter of the 878 respondents (217 or 24.7%) are best characterized by Profile 4. Participants in this profile are likely to describe themselves as being in good health and functioning. They are likely not to be concerned about falls, and they have no problems with leading symptoms. They are also not likely to report difficulties associated with cognition, such as poor memory or concentration. Their reports are consistent with high objective assessments of cognition. Finally, they do not report problems with ADL or IADL tasks. However, Profile 4 neighborhoods, as measured by both self-report and objective indicators, are characterized as having low walkability, unlike those of their healthy peers in Profile 2.

### Risk of Indoor and Outdoor Falls

There are a total of 165 falls reported by 878 respondents (18.8%). Table 3 reports a statistically significant difference in reported falls by type of profile. The percentage of falls is calculated by dividing the number of falls reported by people in a profile by the total number of people in that profile. For example, 32 falls were reported by the 117 people in Profile 1 (27.4%), thus accounting for the greatest percentage of falls. This percentage is followed by Profiles 2, 4, and 3 (17.5, 14.7, and 13.0%, respectively).

**Table 3:** Total number of falls past 6 months by profile.

	Profile 1	Profile 2	Profile 3	Profile 4	Mixed (*n* = 209)	Total (*n* = 878)	*p* Value
	Frail older adults/poor neighborhood walkability (*n* = 117)	Healthy older adults/good neighborhood walkability (*n* = 166)	Cognitively impaired older adults/moderate neighborhood walkability (*n* = 169)	Healthy older adults/poor neighborhood walkability (*n* = 217)			
			
	*n* (%)	*n* (%)	*n* (%)	*n* (%)	*n* (%)	*n* (%)	
All falls (*n* = 165)^a^	32 (27.4)	29 (17.5)	22 (13)	32 (14.7)	50 (23.9)	165 (18.8)	*p* < 0.01
Indoor falls (*n* = 80)	22 (18.8)	10 (6)	12 (7.1)	17 (7.9)	19 (9.1)	80 (9.1)	*p* < 0.01
Outdoor falls (*n* = 83)	10 (8.5)	19 (11.4)	10 (5.9)	14 (6.5)	30 (14.4)	83 (9.5)	*p* = 0.02

*^a^Locations of 2 falls were not identified*.

The location of the fall is strongly associated with characteristics of the profile. Taking into account the number of people in each profile, the percentage reporting an indoor fall ranged from 18.8% in Profile 1 to 6.0% in Profile 2. In contrast, 11.4% of people in Profile 2 reported an outdoor fall, compared to approximately 6% in Profile 3. Among the 208 people who could not be classified in Profile 1, 2, 3, or 4, 9.1% reported an indoor fall and 14.4% reported an outdoor fall. These results indicate that over twice as many of the falls reported by people in Profile 1 occurred indoors than outdoors (18.8 vs. 8.5%). In contrast, more of the falls reported by people in Profile 2 happened outdoors than indoors (11.4 vs. 6.0%). For those in Profile 3, the likelihood of an indoor fall is slightly more common than an outdoor fall (7.1 vs. 5.9%). Finally, unlike the relatively healthy adults in Profile 2, the seemingly healthy people in Profile 4 report slightly more falls occurring indoors than outdoors (7.9 vs. 6.5%).

## Discussion

Profiles 1 and 2 are very similar to the iconic types of fallers described in the current literature: frail older adults who are likely to fall indoors; healthy older adults who are likely to fall outdoors.

Profiles 1 and 2 also provide new information. The neighborhood environment in Profile 1, often ignored in studies of frail seniors at risk of falling, may discourage older adults from spending time outdoors. By spending more time at home, they are more likely to fall there. If Profile 1 respondents do leave their homes, they may be subject to hazards that may elevate the risk for a fall. Compared to the other profiles, Profile 2 is characterized by both the most walkable neighborhoods, based on both self-reported and objective measures; and, interestingly, the greatest proportion of outdoor falls. Walkable neighborhoods by definition encourage walking. If older adults feel at ease, they may be less likely to expect a hazard, such as a broken sidewalk, however rare that might be. Walkable neighborhoods also may encourage a larger number of walkers, thus elevating the risk of a fall from a collision or an attempt to avoid a collision with other pedestrians.

Although Profiles 1 and 2 most closely resemble the iconic indoor and outdoor fallers, these profiles account for less than half of the total number of falls in our sample (37.0%). These results suggest that there may be other sets of risk factors (profiles) beyond the two most commonly reported in the literature. Profiles 3 and 4 suggest new combinations of risk factors not previously reported.

Profile 3 includes older people who are characterized by reasonably good physical health, but limited cognitive function. This may represent a curious combination of “positive” and “negative” factors that may elevate the risk of a fall (e.g., good lower body function and mild cognitive impairment, respectively), previously described by Bergland et al. (3). Future analytic studies should focus on the role of cognitive factors in different neighborhood settings. It is important to note that the respondents in this study were sufficiently cognitively and physically functional to attend a senior center, the site of study selection. Even though a respondent may fall in the lower study distribution of cognitive function, he or she was still quite functional to function independently and complete the interview and direct performance tests.

Profile 4 accounts for almost 25% of the respondents. Unlike respondents in Profile 2, whom they resemble in terms of good health and functioning, those in Profile 4 live in areas of low walkability. Problems with walkability do not seem to be associated with concerns about safety for people in Profile 4, as is the case with Profiles 1 and 3. In fact, respondents in Profile 4 are likely to report that their neighborhoods are safe and free from crime. These respondents are likely to live in suburban areas with low housing density, reduced access to services, and few walking destinations. However, given their relative affluence and reported ease in driving and parking, people in Profile 4 may not be troubled or inconvenienced by goods and services being beyond walking distance. Interestingly, overall falls, especially outdoor falls are more common in Profile 2 than Profile 4, even though the health and functional status of people in both profiles is relatively positive. It may be that walkable environments, as included in Profile 2, may encourage more everyday (“utilitarian”) walking and ironically elevate the risk of an outdoor fall (32).

### Limitations

This is a very promising avenue for research, but there are a number of limitations. First, this is a cross-sectional, descriptive analysis. Although we can identify higher order interactions, it is not possible to specify causal connections. Second, as noted previously, the measure of self-reported falls has been used in other projects there are still significant limitations. Our measure only records whether a respondent has fallen within a 6-month period. It is unclear how many times a respondent may have during that period. We also do not have information about the circumstances of the fall, critical for assessing the interaction with the neighborhood environment.

### New Directions for Prevention

The results support the call to better characterize the heterogeneity of falls (4, 6). A greater appreciation of the heterogeneity of falls should lead to a new set of more targeted and sophisticated prevention strategies. While strategies to improve balance and mobility are important, they are probably not sufficient for all older adults. Including information about the neighborhood context should provide valuable information to refine prevention strategies. GoM seems to represent a promising approach in this regard. New risk profiles may emerge and one or more of the current four profiles may recede or become more precise within the examination of larger and more diverse populations of older adults as well the examination of new risk information. The results of this study, although limited by the variable in the current data set, underscore the value of looking jointly at the intersection of the individual and the environment. For example, although neighborhood walkability has been shown to be associated with the risk of falls (5, 33, 34), there is need for a more systematic and detailed examination of the interplay of neighborhood walkability, the functional capacity of older adults, and the location and circumstances of falls. There is a growing interest in the intersection with neighborhood characteristics (35). Research of this kind may suggest that older adults who live in “walkable environments,” need special attention. With an anticipated increase in the volume and diversity of fellow pedestrians in walkable neighborhoods, older adults may need special instruction in the “rules of the sidewalk,” not unlike ensuring that older drivers are conversant with the “rules of the road.” Of course, this does not preclude direct environmental interventions to install walking and passing lanes to improve safe mobility.

More sophisticated prevention strategies, no doubt, will come from more sophisticated prevention-based research. This may be established in several ways:

1. It is necessary to obtain more detailed information on the timing, location, and circumstances associated with a fall. A life-space approach may be ideally suited for this task (36). This approach is designed to obtain information about a person’s daily activities and movement from the bedroom, to other locations in the house, to the immediate yard and neighborhood, and beyond. Although originally designed to assess and compare the level of mobility of older adults, it is ideally suited to learn more about older adults’ timing, location, and circumstances of regular activities. This information provides a very useful everyday context to then collect information about falls. In this case, we are not highlighting indoor and outdoor falls, but rather the number and location of falls than occur as part of everyday life.
2. While information on life space and falls can be obtained from self-report, it is also necessary to explore the utility of mobile, information technology to unobtrusively monitor the actions of the subjects (37). It is possible to use small wearable devices to monitor unobtrusively mobility and capture, record, and transmit abrupt movements associated with a fall.

In conclusion, research on risk profiles for falls underscores the utility of looking at the intersection of people and places. In addition to improving our understanding of the etiology of different types of falls, research in this area should lead to a new generation of prevention strategies that take into account both people and places.

## Ethics Statement

Informed consent was obtained prior to the interview, as provided by the Institutional Review Board at each of the participating universities: University of California, Berkeley; University of Illinois, Chicago; University of Pittsburgh; and University of North Carolina, Chapel Hill.

## Author Contributions

All authors contributed equally.

## Conflict of Interest Statement

The authors declare that the research was conducted in the absence of any commercial or financial relationships that could be construed as a potential conflict of interest.
