# Supplementary material for: Risk Profiles for Falls among Older Adults: New Directions for Prevention
Source: Front Public Health. 2017 Aug 2;5:142. doi: 10.3389/fpubh.2017.00142 (PMC5539824; doi:10.3389/fpubh.2017.00142)
Supplement: Supplementary file 1 [file Data_Sheet_1.docx]

**Appendix A. Profiles by Individual and Environmental Factors**

|  |  |  |  | **Profile 1** | **Profile 2** | **Profile 3** | **Profile 4** |
| --- | --- | --- | --- | --- | --- | --- | --- |
| **Variable** | **Category^a^** | **N** | **%** | **P(P1)^b^** | **P(P2)^c^** | **P(P3)^d^** | **P(P4)^e^** |
| **Age (years)** | 65-74 | 451 | 51.00 | 0.2707 | 0.4074 | 0.5736 | 0.7229 |
|  | 75+ | 433 | 49.00 | 0.7293 | 0.5926 | 0.4264 | 0.2771 |
|  |  |  |  |  |  |  |  |
| **Overall health** | Excellent | 231 | 26.10 | 0.0000 | **0.5992** | 0.0000 | 0.4131 |
|  | Good | 459 | 51.90 | 0.1933 | 0.4008 | 0.8788 | 0.5594 |
|  | Fair/poor | 194 | 21.90 | **0.8067** | 0.0000 | 0.1212 | 0.0275 |
|  |  |  |  |  |  |  |  |
| **Sex** | Male | 207 | 23.40 | 0.0642 | **0.5797** | 0.0660 | 0.1890 |
|  | Female | 677 | 76.60 | 0.9358 | 0.4203 | 0.9340 | 0.8110 |
|  |  |  |  |  |  |  |  |
| **Health compared to others** | Better | 523 | 59.20 | 0.1406 | **1.0000** | 0.4038 | 0.7348 |
|  | Same | 329 | 37.20 | **0.7285** | 0.0000 | 0.5699 | 0.2543 |
|  | Worse | 26 | 2.90 | **0.1309** | 0.0000 | 0.0000 | 0.0108 |
|  | Missing | 6 | 0.70 | 0.0000 | 0.0000 | **0.0263** | 0.0000 |
|  |  |  |  |  |  |  |  |
| **Need glasses/lenses** | Yes | 795 | 89.90 | 0.9171 | 0.8807 | 0.8919 | 0.9144 |
|  | No | 88 | 10.00 | 0.0829 | 0.1193 | 0.1081 | 0.0856 |
|  | Missing | 1 | 0.10 | 0.0000 | 0.0000 | 0.0000 | 0.0000 |

|  |  |  |  | **Profile 1** | **Profile 2** | **Profile 3** | **Profile 4** |
| --- | --- | --- | --- | --- | --- | --- | --- |
| **Variable** | **Category^a^** | **N** | **%** | **P(P1)^b^** | **P(P2)^c^** | **P(P3)^d^** | **P(P4)^e^** |
| **Eyesight compared to others** | Better | 284 | 32.10 | 0.2646 | 0.5273 | 0.1049 | 0.3707 |
|  | Same | 524 | 59.30 | 0.5790 | 0.3104 | 0.8875 | 0.5898 |
|  | Worse | 65 | 7.40 | **0.1474** | 0.1209 | 0.0077 | 0.0395 |
|  | Missing | 11 | 1.20 | 0.0090 | **0.0414** | 0.0000 | 0.0000 |
|  |  |  |  |  |  |  |  |
| **Hearing compared to**  **Others** | Better | 90 | 10.20 | **0.2065** | 0.0195 | 0.0592 | 0.1416 |
|  | Same | 482 | 54.50 | 0.4757 | 0.4605 | 0.6637 | 0.5685 |
|  | Worse | 295 | 33.40 | 0.2920 | 0.4718 | 0.2702 | 0.2899 |
|  | Missing | 17 | 1.90 | 0.0257 | **0.0482** | 0.0069 | 0.0000 |
|  |  |  |  |  |  |  |  |
| **Limit or avoid activities because of concern about falls** | Yes | 119 | 13.50 | **0.6136** | 0.0316 | 0.0000 | 0.0000 |
|  | No | 765 | 86.50 | 0.3864 | 0.9684 | **1.0000** | **1.0000** |
|  |  |  |  |  |  |  |  |
| **Total walking (mins/wk)** | <150 | 543 | 61.40 | 0.9612 | 0.5133 | 0.6075 | 0.4542 |
|  | 150+ | 334 | 37.80 | 0.0388 | 0.4867 | 0.3617 | 0.5458 |
|  | Missing | 7 | 0.80 | 0.0000 | 0.0000 | 0.0307 | 0.0000 |
|  |  |  |  |  |  |  |  |
| **Total number of functional limitations** | None | 148 | 16.70 | 0.0000 | **0.3278** | 0.0663 | 0.2717 |
|  | 1-2 | 226 | 25.60 | 0.0000 | 0.1691 | 0.3074 | 0.5556 |
|  | 3-5 | 234 | 26.50 | 0.0090 | 0.4354 | 0.4737 | 0.1727 |

|  |  |  |  | **Profile 1** | **Profile 2** | **Profile 3** | **Profile 4** |
| --- | --- | --- | --- | --- | --- | --- | --- |
| **Variable** | **Category^a^** | **N** | **%** | **P(P1)^b^** | **P(P2)^c^** | **P(P3)^d^** | **P(P4)^e^** |
| **Total number of functional limitations (cont’d)** | >5 | 276 | 31.20 | **0.9910** | 0.0677 | 0.1526 | 0.0000 |
|  |  |  |  |  |  |  |  |
| **ADLs^f^, assistance needed** | None | 803 | 90.80 | 0.5420 | **1.0000** | **1.0000** | **1.0000** |
|  | 1 task | 48 | 5.40 | **0.2750** | 0.0000 | 0.0000 | 0.0000 |
|  | >1 task | 32 | 3.60 | **0.1830** | 0.0000 | 0.0000 | 0.0000 |
|  | Missing | 1 | 0.10 | 0.0000 | 0.0000 | 0.0000 | 0.0000 |
|  |  |  |  |  |  |  |  |
| **Difficulty walking due to leg weakness** | Yes | 141 | 16.00 | **0.9133** | 0.0000 | 0.0000 | 0.0000 |
|  | No | 742 | 83.90 | 0.0867 | **1.0000** | **1.0000** | **1.0000** |
|  | Missing | 1 | 0.10 | 0.0000 | 0.0000 | 0.0000 | 0.0000 |
|  |  |  |  |  |  |  |  |
| **Difficulty walking due to leg pain** | Yes | 273 | 30.90 | **1.0000** | 0.1785 | 0.0095 | 0.0528 |
|  | No | 610 | 69.00 | 0.0000 | 0.8215 | 0.9905 | 0.9472 |
|  | Missing | 1 | 0.10 | 0.0000 | 0.0000 | 0.0000 | 0.0000 |
|  |  |  |  |  |  |  |  |
| **Difficulty walking due to back pain** | Yes | 155 | 17.50 | **0.8256** | 0.0000 | 0.0000 | 0.0000 |
|  | No | 723 | 81.80 | 0.1744 | **1.0000** | **1.0000** | **1.0000** |
|  | Missing | 6 | 0.70 | 0.0000 | 0.0000 | 0.0000 | 0.0000 |
|  |  |  |  |  |  |  |  |
| **Difficulty walking due to problem standing tall** | Yes | 84 | 9.50 | **0.4868** | 0.0000 | 0.0000 | 0.0000 |
|  | No | 794 | 89.80 | 0.4829 | 0.9970 | **1.0000** | **1.0000** |

|  |  |  |  | **Profile 1** | **Profile 2** | **Profile 3** | **Profile 4** |
| --- | --- | --- | --- | --- | --- | --- | --- |
| **Variable** | **Category^a^** | **N** | **%** | **P(P1)^b^** | **P(P2)^c^** | **P(P3)^d^** | **P(P4)^e^** |
| **Difficulty walking due to problem standing tall (cont’d)** | Missing | 6 | 0.70 | 0.0302 | 0.0030 | 0.0000 | 0.0000 |
|  |  |  |  |  |  |  |  |
| **Difficulty walking due to neck pain** | Yes | 57 | 6.40 | **0.3270** | 0.0000 | 0.0000 | 0.0000 |
|  | No | 824 | 93.20 | 0.6616 | **1.0000** | **1.0000** | 0.9944 |
|  | Missing | 3 | 0.30 | **0.0114** | 0.0000 | 0.0000 | **0.0056** |
|  |  |  |  |  |  |  |  |
| **Difficulty walking due to problem with memory** | Yes | 62 | 7.00 | **0.3292** | 0.0124 | 0.0000 | 0.0000 |
|  | No | 822 | 93.00 | 0.6708 | 0.9876 | **1.0000** | **1.0000** |
|  |  |  |  |  |  |  |  |
| **Difficulty walking due to problem with concentration** | Yes | 32 | 3.60 | **0.1815** | 0.0000 | 0.0000 | 0.0000 |
|  | No | 847 | 95.80 | 0.8014 | **1.0000** | 0.9912 | **1.0000** |
|  | Missing | 5 | 0.60 | **0.0171** | 0.0000 | 0.0088 | 0.0000 |
|  |  |  |  |  |  |  |  |
| **Difficulty walking due to problem with seeing steps** | Yes | 52 | 5.90 | **0.2923** | 0.0000 | 0.0000 | 0.0000 |
|  | No | 826 | 93.40 | 0.6735 | **1.0000** | **1.0000** | **1.0000** |
|  | Missing | 6 | 0.70 | **0.0342** | 0.0000 | 0.0000 | 0.0000 |
|  |  |  |  |  |  |  |  |
| **Difficulty walking due to problem with glare** | Yes | 148 | 16.70 | **0.6410** | 0.1532 | 0.0000 | 0.0000 |
|  | No | 734 | 83.00 | 0.3590 | 0.8468 | **1.0000** | **1.0000** |
|  | Missing | 2 | 0.20 | 0.0000 | 0.0000 | 0.0000 | 0.0000 |

|  |  |  |  | **Profile 1** | **Profile 2** | **Profile 3** | **Profile 4** |
| --- | --- | --- | --- | --- | --- | --- | --- |
| **Variable** | **Category^a^** | **N** | **%** | **P(P1)^b^** | **P(P2)^c^** | **P(P3)^d^** | **P(P4)^e^** |
| **Difficulty walking due to dizziness** | Yes | 68 | 7.70 | **0.3881** | 0.0000 | 0.0000 | 0.0000 |
|  | No | 813 | 92.00 | 0.6119 | **1.0000** | **1.0000** | **1.0000** |
|  | Missing | 3 | 0.30 | 0.0000 | 0.0000 | 0.0000 | 0.0000 |
|  |  |  |  |  |  |  |  |
| **Difficulty walking due to problem with balance** | Yes | 123 | 13.90 | **0.7074** | 0.0000 | 0.0000 | 0.0000 |
|  | No | 756 | 85.50 | 0.2750 | 0.9917 | **1.0000** | **1.0000** |
|  | Missing | 5 | 0.60 | **0.0177** | 0.0083 | 0.0000 | 0.0000 |
|  |  |  |  |  |  |  |  |
| **Difficulty walking due to need to use bathroom** | Yes | 108 | 12.20 | **0.5903** | 0.0000 | 0.0000 | 0.0000 |
|  | No | 770 | 87.10 | 0.3755 | **1.0000** | **1.0000** | **1.0000** |
|  | Missing | 6 | 0.70 | **0.0341** | 0.0000 | 0.0000 | 0.0000 |
|  |  |  |  |  |  |  |  |
| **Difficulty walking due to fatigue** | Yes | 167 | 18.90 | **0.8528** | 0.0000 | 0.0000 | 0.0000 |
|  | No | 711 | 80.40 | 0.1131 | **1.0000** | **1.0000** | **1.0000** |
|  | Missing | 6 | 0.70 | **0.0341** | 0.0000 | 0.0000 | 0.0000 |
|  |  |  |  |  |  |  |  |
| **Difficulty walking due to problem with start/stops** | Yes | 30 | 3.40 | **0.1711** | 0.0000 | 0.0000 | 0.0000 |
|  | No | 849 | 96.00 | 0.8289 | **1.0000** | **1.0000** | **1.0000** |
|  | Missing | 5 | 0.60 | 0.0000 | 0.0000 | 0.0000 | 0.0000 |

|  |  |  |  | **Profile 1** | **Profile 2** | **Profile 3** | **Profile 4** |
| --- | --- | --- | --- | --- | --- | --- | --- |
| **Variable** | **Category^a^** | **N** | **%** | **P(P1)^b^** | **P(P2)^c^** | **P(P3)^d^** | **P(P4)^e^** |
| **Difficulty walking due to chest pain** | Yes | 27 | 3.10 | **0.1534** | 0.0000 | 0.0000 | 0.0000 |
|  | No | 856 | 96.80 | 0.8466 | **1.0000** | **1.0000** | **1.0000** |
|  | Missing | 1 | 0.10 | 0.0000 | 0.0000 | 0.0000 | 0.0000 |
|  |  |  |  |  |  |  |  |
| **Difficulty walking due to shortness of breath** | Yes | 120 | 13.60 | **0.6604** | 0.0000 | 0.0000 | 0.0000 |
|  | No | 759 | 85.90 | 0.3226 | **1.0000** | 0.9911 | **1.0000** |
|  | Missing | 5 | 0.60 | 0.0170 | 0.0000 | 0.0089 | 0.0000 |
|  |  |  |  |  |  |  |  |
| **Worry about falls** | Never | 455 | 51.50 | 0.0003 | 0.6330 | 0.5934 | 0.7257 |
|  | Little | 209 | 23.60 | 0.2070 | 0.3140 | 0.2184 | 0.2046 |
|  | Some | 135 | 15.30 | **0.3787** | 0.0530 | 0.1632 | 0.0697 |
|  | A lot | 83 | 9.40 | **0.4140** | 0.0000 | 0.0250 | 0.0000 |
|  | Missing | 2 | 0.20 | 0.0000 | 0.0000 | 0.0000 | 0.0000 |
|  |  |  |  |  |  |  |  |
| **MMSE^g^ quartiles** | Low | 236 | 26.70 | **0.5303** | 0.0000 | **0.5321** | 0.0615 |
|  | Somewhat low | 311 | 35.20 | 0.3347 | 0.4012 | 0.3393 | 0.3282 |
|  | Somewhat high | 140 | 15.80 | 0.0540 | **0.3683** | 0.0000 | 0.1837 |
|  | High | 148 | 16.70 | 0.0059 | 0.2305 | 0.0000 | **0.4027** |
|  | Missing | 49 | 5.50 | 0.0752 | 0.0000 | **0.1286** | 0.0239 |

|  |  |  |  | **Profile 1** | **Profile 2** | **Profile 3** | **Profile 4** |
| --- | --- | --- | --- | --- | --- | --- | --- |
| **Variable** | **Category^a^** | **N** | **%** | **P(P1)^b^** | **P(P2)^c^** | **P(P3)^d^** | **P(P4)^e^** |
| **MAT^h^ quartiles** | Low | 234 | 26.50 | 0.4229 | 0.0000 | **0.4780** | 0.1992 |
|  | Somewhat low | 237 | 26.80 | 0.2355 | 0.0357 | 0.4690 | 0.3233 |
|  | Somewhat high | 205 | 23.20 | 0.1934 | 0.4019 | 0.0530 | 0.2736 |
|  | High | 199 | 22.50 | 0.1146 | **0.5486** | 0.0000 | 0.2039 |
|  | Missing | 9 | 1.00 | **0.0335** | 0.0138 | 0.0000 | 0.0000 |
|  |  |  |  |  |  |  |  |
| **Depressed (CES-D^i^≥10)** | Depressed | 133 | 15.00 | **0.5177** | 0.0000 | 0.1932 | 0.0000 |
|  | Not depressed | 737 | 83.40 | 0.4379 | **1.0000** | 0.7873 | 0.9924 |
|  | Missing | 14 | 1.60 | **0.0444** | 0.0000 | 0.0195 | 0.0076 |
|  |  |  |  |  |  |  |  |
| **Confidence walking 10 blocks** | Low | 249 | 28.20 | **0.9897** | 0.0000 | 0.0292 | 0.0000 |
|  | Somewhat low | 216 | 24.40 | 0.0103 | 0.2020 | **0.5431** | 0.2197 |
|  | Somewhat high | 418 | 47.30 | 0.0000 | 0.7980 | 0.4276 | 0.7803 |
|  | Missing | 1 | 0.10 | 0.0000 | 0.0000 | 0.0000 | 0.0000 |
|  |  |  |  |  |  |  |  |
| **Spouse** | Yes | 340 | 38.50 | 0.0234 | **0.6475** | 0.0341 | **0.7352** |
|  | No | 543 | 61.40 | 0.9766 | 0.3525 | 0.9659 | 0.2648 |
|  | Missing | 1 | 0.10 | 0.0000 | 0.0000 | 0.0000 | 0.0000 |
|  |  |  |  |  |  |  |  |
| **IADLs^j^, limitations/3 tasks** | None | 767 | 86.80 | 0.4069 | 0.9882 | 0.9517 | **1.0000** |
|  | 1 | 68 | 7.70 | **0.3416** | 0.0000 | 0.0388 | 0.0000 |
|  | 2 | 15 | 1.70 | **0.0855** | 0.0000 | 0.0000 | 0.0000 |
|  |  |  |  | **Profile 1** | **Profile 2** | **Profile 3** | **Profile 4** |
| **Variable** | **Category^a^** | **N** | **%** | **P(P1)^b^** | **P(P2)^c^** | **P(P3)^d^** | **P(P4)^e^** |
| **IADLs^j^, limitations/3 tasks (cont’d)** | All 3 | 22 | 2.50 | **0.1253** | 0.0000 | 0.0000 | 0.0000 |
|  | Missing | 12 | 1.40 | **0.0408** | 0.0118 | 0.0095 | 0.0000 |
|  |  |  |  |  |  |  |  |
| **NEWS^k^ walk time to most common destinations** | Short | 220 | 24.90 | 0.0000 | **0.5871** | 0.3371 | 0.0000 |
|  | Somewhat short | 231 | 26.10 | 0.1263 | 0.4129 | **0.4742** | 0.0425 |
|  | Somewhat long | 182 | 20.60 | 0.3053 | 0.0000 | 0.1887 | 0.3469 |
|  | Long | 251 | 28.40 | **0.5684** | 0.0000 | 0.0000 | **0.6106** |
|  |  |  |  |  |  |  |  |
| **NEWS^k^ neighborhood buildings** | Residential | 728 | 82.40 | 0.9010 | 0.8550 | 0.5420 | **1.0000** |
|  | Commercial | 155 | 17.50 | 0.0990 | 0.1450 | **0.4580** | 0.0000 |
|  | Missing | 1 | 0.10 | 0.0000 | 0.0000 | 0.0000 | 0.0000 |
|  |  |  |  |  |  |  |  |
| **NEWS^k^ housing in neighborhood** | Single family | 512 | 57.90 | 0.4468 | 0.6573 | 0.1577 | 0.9954 |
|  | Mixed single family/apt | 267 | 30.20 | 0.3236 | 0.2968 | **0.6031** | 0.0046 |
|  | Apt/condo | 96 | 10.90 | 0.1781 | 0.0459 | **0.2392** | 0.0000 |
|  | Missing | 9 | 1.00 | **0.0516** | 0.0000 | 0.0000 | 0.0000 |
|  |  |  |  |  |  |  |  |
| **NEWS^k^ land use/walking accessibility to services** | Low accessibility | 293 | 33.10 | 0.4963 | 0.0000 | 0.0000 | **0.8459** |
|  | Somewhat low | 217 | 24.50 | 0.3353 | 0.0709 | **0.4936** | 0.1541 |
|  | Somewhat high | 181 | 20.50 | 0.1341 | 0.3389 | 0.3359 | 0.0000 |

|  |  |  |  | **Profile 1** | **Profile 2** | **Profile 3** | **Profile 4** |
| --- | --- | --- | --- | --- | --- | --- | --- |
| **Variable** | **Category^a^** | **N** | **%** | **P(P1)^b^** | **P(P2)^c^** | **P(P3)^d^** | **P(P4)^e^** |
| **NEWS^k^ land use/walking accessibility to services (cont’d)** | High | 187 | 21.20 | 0.0000 | **0.5901** | 0.1705 | 0.0000 |
|  | Missing | 6 | 0.70 | 0.0343 | 0.0000 | 0.0000 | 0.0000 |
|  |  |  |  |  |  |  |  |
| **NEWS^k^ street connectivity** | Low | 298 | 33.70 | 0.4500 | 0.0000 | 0.2180 | **0.6582** |
|  | Somewhat low | 177 | 20.00 | 0.3361 | 0.0937 | 0.2622 | 0.1499 |
|  | Somewhat high | 399 | 45.10 | 0.1565 | **0.9063** | 0.5199 | 0.1920 |
|  | Missing | 10 | 1.10 | **0.0574** | 0.0000 | 0.0000 | 0.0000 |
|  |  |  |  |  |  |  |  |
| **NEWS^k^ quality of walking places** | Poor | 282 | 31.90 | **0.6096** | 0.0000 | 0.0000 | **0.7255** |
|  | Somewhat poor | 202 | 22.90 | 0.1593 | 0.0766 | **0.5930** | 0.1571 |
|  | Somewhat good | 213 | 24.10 | 0.2311 | 0.2832 | 0.3545 | 0.1173 |
|  | Good | 179 | 20.20 | 0.0000 | **0.6401** | 0.0525 | 0.0000 |
|  | Missing | 8 | 0.90 | 0.0000 | 0.0000 | 0.0000 | 0.0000 |
|  |  |  |  |  |  |  |  |
| **NEWS^k^ attractiveness of surroundings** | Not attractive | 223 | 25.20 | **0.6807** | 0.0000 | 0.4287 | 0.0819 |
|  | Somewhat not attractive | 268 | 30.30 | 0.1269 | 0.1828 | 0.5020 | 0.3482 |
|  | Somewhat attractive | 176 | 19.90 | 0.0894 | **0.4034** | 0.0693 | 0.1850 |
|  | Attractive | 209 | 23.60 | 0.1029 | 0.4138 | 0.0000 | 0.3849 |
|  | Missing | 8 | 0.90 | 0.0000 | 0.0000 | 0.0000 | 0.0000 |

|  |  |  |  | **Profile 1** | **Profile 2** | **Profile 3** | **Profile 4** |
| --- | --- | --- | --- | --- | --- | --- | --- |
| **Variable** | **Category^a^** | **N** | **%** | **P(P1)^b^** | **P(P2)^c^** | **P(P3)^d^** | **P(P4)^e^** |
| **NEWS^k^ traffic safety** | Poor | 282 | 31.90 | 0.5686 | 0.0458 | **0.5676** | 0.1580 |
|  | Fair | 231 | 26.10 | 0.2759 | 0.3465 | 0.2896 | 0.1475 |
|  | Good | 163 | 18.40 | 0.1041 | 0.1906 | 0.1427 | 0.2736 |
|  | Excellent | 199 | 22.50 | 0.0000 | **0.4171** | 0.0000 | **0.4209** |
|  | Missing | 9 | 1.00 | **0.0513** | 0.0000 | 0.0000 | 0.0000 |
|  |  |  |  |  |  |  |  |
| **NEWS^k^ crime safety** | Unsafe | 162 | 18.30 | **0.3881** | 0.0000 | **0.4263** | 0.0000 |
|  | Somewhat safe | 211 | 23.90 | 0.2879 | 0.1364 | **0.5737** | 0.0000 |
|  | Safe | 493 | 55.80 | 0.2189 | 0.8636 | 0.0000 | **1.0000** |
|  | Missing | 18 | 2.00 | **0.1051** | 0.0000 | 0.0000 | 0.0000 |
|  |  |  |  |  |  |  |  |
| **NEWS^k^ neighborhood**  **satisfaction/residents know**  **each other** | Don't know each other | 275 | 31.10 | 0.4805 | 0.0300 | **0.5656** | 0.2139 |
|  | Somewhat don't know each other | 282 | 31.90 | 0.2498 | 0.2526 | 0.3222 | 0.4216 |
|  | Somewhat know each other | 104 | 11.80 | 0.0667 | **0.3088** | 0.0287 | 0.0621 |
|  | Know each other | 213 | 24.10 | 0.1460 | 0.4086 | 0.0835 | 0.3023 |
|  | Missing | 10 | 1.10 | **0.0570** | 0.0000 | 0.0000 | 0.0000 |

|  |  |  |  | **Profile 1** | **Profile 2** | **Profile 3** | **Profile 4** |
| --- | --- | --- | --- | --- | --- | --- | --- |
| **Variable** | **Category^a^** | **N** | **%** | **P(P1)^b^** | **P(P2)^c^** | **P(P3)^d^** | **P(P4)^e^** |
| **NEWS^k^ difficulty parking** | Easy | 353 | 39.90 | 0.1622 | 0.5377 | 0.0000 | **0.8028** |
|  | Somewhat easy | 163 | 18.40 | 0.2121 | 0.0838 | 0.3173 | 0.1330 |
|  | Somewhat difficult | 191 | 21.60 | 0.3036 | 0.2942 | 0.2550 | 0.0530 |
|  | Difficult | 116 | 13.10 | 0.1940 | 0.0796 | **0.2648** | 0.0112 |
|  | Missing | 61 | 6.90 | **0.1281** | 0.0047 | **0.1628** | 0.0000 |
|  |  |  |  |  |  |  |  |
| **NEWS^k^ cul-de-sacs** | Lots | 190 | 21.50 | 0.1913 | 0.1489 | 0.1000 | **0.3946** |
|  | Some | 127 | 14.40 | 0.1054 | 0.0000 | 0.2114 | 0.2382 |
|  | Few | 165 | 18.70 | 0.2383 | 0.0000 | 0.3343 | 0.1874 |
|  | None | 384 | 43.40 | 0.3814 | 0.8511 | 0.3395 | 0.1798 |
|  | Missing | 18 | 2.00 | **0.0836** | 0.0000 | 0.0148 | 0.0000 |
|  |  |  |  |  |  |  |  |
| **NEWS^k^ street grade** | Hilly | 137 | 15.50 | 0.2437 | 0.0000 | 0.1964 | 0.2039 |
|  | Somewhat hilly | 145 | 16.40 | 0.1482 | 0.0404 | 0.2609 | 0.2009 |
|  | Somewhat flat | 120 | 13.60 | 0.1387 | 0.0761 | 0.1898 | 0.1402 |
|  | Flat | 468 | 52.90 | 0.3899 | 0.8835 | 0.3515 | 0.4550 |
|  | Missing | 14 | 1.60 | **0.0795** | 0.0000 | 0.0014 | 0.0000 |

|  |  |  |  | **Profile 1** | **Profile 2** | **Profile 3** | **Profile 4** |
| --- | --- | --- | --- | --- | --- | --- | --- |
| **Variable** | **Category^a^** | **N** | **%** | **P(P1)^b^** | **P(P2)^c^** | **P(P3)^d^** | **P(P4)^e^** |
| **NEWS^k^ walking barriers** | Major | 63 | 7.10 | **0.1448** | 0.0000 | 0.0740 | 0.0825 |
|  | Somewhat major | 70 | 7.90 | 0.0954 | 0.0000 | 0.1196 | 0.1030 |
|  | Few | 140 | 15.80 | 0.2761 | 0.0000 | **0.3266** | 0.0668 |
|  | None | 601 | 68.00 | 0.4267 | **1.0000** | 0.4798 | 0.7477 |
|  | Missing | 10 | 1.10 | **0.0570** | 0.0000 | 0.0000 | 0.0000 |
|  |  |  |  |  |  |  |  |
| **Access to vehicle** | Yes | 726 | 82.10 | 0.6460 | **1.0000** | 0.5951 | **1.0000** |
|  | No | 150 | 17.00 | **0.3083** | 0.0000 | **0.4049** | 0.0000 |
|  | Missing | 8 | 0.90 | **0.0456** | 0.0000 | 0.0000 | 0.0000 |
|  |  |  |  |  |  |  |  |
| **Financial needs met** | Not adequately | 58 | 6.60 | **0.2250** | 0.0000 | 0.0826 | 0.0000 |
|  | Somewhat adequately | 367 | 41.50 | 0.6700 | 0.0672 | **0.8699** | 0.1160 |
|  | Very adequately | 444 | 50.20 | 0.0851 | **0.9328** | 0.0433 | 0.8419 |
|  | Missing | 15 | 1.70 | 0.0200 | 0.0000 | 0.0043 | **0.0422** |
|  |  |  |  |  |  |  |  |
| **Income** | <$15,000 | 177 | 20.00 | **0.4714** | 0.0000 | **0.3766** | 0.0000 |
|  | $15,000-24,999 | 163 | 18.40 | 0.2153 | 0.1677 | 0.2838 | 0.0693 |
|  | $25,000-49,999 | 203 | 23.00 | 0.0927 | **0.5507** | 0.0000 | 0.2902 |
|  | $50,000+ | 137 | 15.50 | 0.0000 | 0.1876 | 0.0000 | **0.3945** |

|  |  |  |  | **Profile 1** | **Profile 2** | **Profile 3** | **Profile 4** |
| --- | --- | --- | --- | --- | --- | --- | --- |
| **Variable** | **Category^a^** | **N** | **%** | **P(P1)^b^** | **P(P2)^c^** | **P(P3)^d^** | **P(P4)^e^** |
| **Income (cont’d)** | Refused/don’t know | 196 | 22.20 | 0.1747 | 0.0939 | 0.3396 | 0.2459 |
|  | Missing | 8 | 0.90 | **0.0458** | 0.0000 | 0.0000 | 0.0000 |
|  |  |  |  |  |  |  |  |
| **Employment** | Yes | 89 | 10.10 | 0.1114 | **0.2245** | 0.0000 | 0.0780 |
|  | No | 786 | 88.90 | 0.8373 | 0.7755 | **1.0000** | 0.9220 |
|  | Missing | 9 | 1.00 | **0.0512** | 0.0000 | 0.0000 | 0.0000 |
|  |  |  |  |  |  |  |  |
| **Volunteerism** | Yes | 460 | 52.00 | 0.3814 | 0.6644 | 0.4752 | 0.5299 |
|  | No | 417 | 47.20 | 0.5787 | 0.3356 | 0.5248 | 0.4701 |
|  | Missing | 7 | 0.80 | **0.0399** | 0.0000 | 0.0000 | 0.0000 |
|  |  |  |  |  |  |  |  |
| **Service as caregiver** | Yes | 71 | 8.00 | 0.0555 | **0.1486** | 0.0631 | 0.0561 |
|  | No | 804 | 91.00 | 0.8933 | 0.8514 | 0.9369 | 0.9439 |
|  | Missing | 9 | 1.00 | **0.0512** | 0.0000 | 0.0000 | 0.0000 |
|  |  |  |  |  |  |  |  |
| **Education** | 0-11 | 104 | 11.80 | **0.2853** | 0.0000 | **0.2448** | 0.0000 |
|  | 12 | 284 | 32.10 | 0.4215 | 0.1229 | **0.5949** | 0.1904 |
|  | >12 | 484 | 54.80 | 0.2242 | 0.8771 | 0.1603 | 0.8096 |
|  | Missing | 12 | 1.40 | **0.0689** | 0.0000 | 0.0000 | 0.0000 |

|  |  |  |  | **Profile 1** | **Profile 2** | **Profile 3** | **Profile 4** |
| --- | --- | --- | --- | --- | --- | --- | --- |
| **Variable** | **Category^a^** | **N** | **%** | **P(P1)^b^** | **P(P2)^c^** | **P(P3)^d^** | **P(P4)^e^** |
| **Race/ethnicity** | White | 577 | 65.30 | 0.4401 | 0.8898 | 0.3524 | 0.8657 |
|  | African American | 206 | 23.30 | **0.4602** | 0.0000 | **0.5401** | 0.0000 |
|  | Asian | 54 | 6.10 | 0.0089 | 0.1020 | 0.0000 | **0.1161** |
|  | Other/1 race | 14 | 1.60 | 0.0000 | 0.0000 | **0.0547** | 0.0057 |
|  | Other/2+races | 16 | 1.80 | 0.0224 | 0.0000 | **0.0528** | 0.0000 |
|  | Missing | 17 | 1.90 | **0.0684** | 0.0082 | 0.0000 | 0.0125 |
|  |  |  |  |  |  |  |  |
| **GIS^l^ housing density quartiles** | Least | 214 | 24.20 | 0.0073 | 0.0000 | 0.0000 | **0.7292** |
|  | Less | 215 | 24.30 | 0.3350 | 0.4032 | 0.0000 | 0.2708 |
|  | More | 211 | 23.90 | **0.4582** | 0.2776 | 0.3592 | 0.0000 |
|  | Most | 216 | 24.40 | 0.1995 | 0.3192 | **0.5164** | 0.0000 |
|  | Missing | 28 | 3.20 | 0.0000 | 0.0000 | **0.1244** | 0.0000 |
|  |  |  |  |  |  |  |  |
| **GIS^l^ median block length** | Shortest | 208 | 23.50 | 0.4030 | 0.0000 | **0.7458** | 0.0000 |
|  | Short | 209 | 23.60 | 0.2607 | **0.6898** | 0.0104 | 0.0000 |
|  | Long | 208 | 23.50 | 0.2992 | 0.2466 | 0.1413 | 0.2751 |
|  | Longest | 208 | 23.50 | 0.0000 | 0.0000 | 0.0000 | **0.6975** |
|  | Missing | 51 | 5.80 | 0.0371 | 0.0636 | 0.1025 | 0.0274 |
|  |  |  |  |  |  |  |  |
| **GIS^l^ count of businesses within 400m of residence** | Few | 277 | 31.30 | 0.0000 | 0.0000 | 0.0000 | **0.8733** |
|  | Somewhat few | 175 | 19.80 | **0.3583** | 0.2655 | 0.1502 | 0.1267 |

|  |  |  |  | **Profile 1** | **Profile 2** | **Profile 3** | **Profile 4** |
| --- | --- | --- | --- | --- | --- | --- | --- |
| **Variable** | **Category^a^** | **N** | **%** | **P(P1)^b^** | **P(P2)^c^** | **P(P3)^d^** | **P(P4)^e^** |
| **GIS^l^ count of businesses within 400m of residence (cont’d)** | More | 193 | 21.80 | **0.4546** | 0.1736 | **0.4166** | 0.0000 |
|  | Many | 212 | 24.00 | 0.1870 | 0.5609 | 0.3128 | 0.0000 |
|  | Missing | 27 | 3.10 | 0.0000 | 0.0000 | **0.1204** | 0.0000 |
|  |  |  |  |  |  |  |  |
| **Lower body functioning^m^** | Lowest | 296 | 33.50 | **0.8264** | 0.0767 | 0.3837 | 0.0718 |
|  | Somewhat low | 271 | 30.70 | 0.0461 | 0.4162 | 0.4049 | 0.3600 |
|  | Somewhat high | 140 | 15.80 | 0.0000 | **0.3097** | 0.1158 | 0.1862 |
|  | High | 150 | 17.00 | 0.0000 | 0.1806 | 0.0955 | 0.3821 |
|  | Missing | 27 | 3.10 | **0.1275** | 0.0169 | 0.0000 | 0.0000 |
|  |  |  |  |  |  |  |  |
| **Years at address** | <10 | 304 | 34.40 | 0.3700 | 0.2148 | 0.5008 | 0.3408 |
|  | 20-30 | 128 | 14.50 | 0.1691 | 0.1702 | 0.0513 | 0.1961 |
|  | >20-30 | 110 | 12.40 | 0.1111 | 0.0632 | 0.1425 | 0.1768 |
|  | >30-40 | 149 | 16.90 | 0.1364 | 0.2570 | 0.1671 | 0.1237 |
|  | >40-50 | 119 | 13.50 | 0.0607 | 0.1635 | 0.1384 | 0.1624 |
|  | >50-60 | 56 | 6.30 | **0.1527** | **0.1313** | 0.0000 | 0.0000 |
|  | >60 | 18 | 2.00 | 0.0000 | 0.0000 | 0.0000 | 0.0000 |

|  |  |  |  | **Profile 1** | **Profile 2** | **Profile 3** | **Profile 4** |
| --- | --- | --- | --- | --- | --- | --- | --- |
| **Variable** | **Category^a^** | **N** | **%** | **P(P1)^b^** | **P(P2)^c^** | **P(P3)^d^** | **P(P4)^e^** |
| **Number close friends and/or relatives** | 0-3 | 236 | 26.70 | 0.3433 | 0.0000 | **0.6469** | 0.0883 |
|  | 4-5 | 209 | 23.60 | 0.2735 | 0.2546 | 0.1482 | 0.2813 |
|  | 6-10 | 244 | 27.60 | 0.3351 | 0.3461 | 0.1531 | 0.2930 |
|  | 11-100 | 172 | 19.50 | 0.0000 | **0.3994** | 0.0000 | 0.3263 |
|  | Missing | 23 | 2.60 | **0.0481** | 0.0000 | **0.0518** | 0.0110 |

^a^Distinguishing category is 1.8 times its probability of being in the profile. Distinguishing categories are bolded in profiles.

^b^Frail Older Adults/Poor Neighborhood Walkability

^c^Healthy Older Adults/Good Neighborhood Walkability

^d^Cognitively Impaired Older Adults/Moderate Neighborhood Walkability

^e^Healthy Older Adults/Poor Neighborhood Walkability

^f^Activities of Daily Living

^g^Modified Mini-Mental State Exam

^h^Mental Alternation Test

^i^Center for Epidemiologic Studies Depression Scale

^j^Instrumental Activities of Daily Living

**^k^**Neighborhood Environment Walkability Scale

^l^ Geographic Information System

**^m^**Summary of direct measures of balance, walking speed and lower-body strength

References

Andreotti, A., Minicuci, N., Kowal, P., & Chatterji, S. (2009). Multidimensional profiles of health status: An application of the Grade of Membership Model to the World Health Survey. *PloS One, 4*(2), e4426.

Andresen, E. M., Malmgren, J. A., Carter, W. B., & Patrick, D. L. (1994). Screening for depression in well older adults: evaluation of a short form of the CES-D (Center for Epidemiologic Studies Depression Scale). *Am J Prev Med, 10*(2), 77-84.

Barnes, D. E., Yaffe, K., Satariano, W. A., & Tager, I. B. (2003). A longitudinal study of cardiorespiratory fitness and cognitive function in healthy older adults. *J Am Geriatr Soc, 51*(4), 459-465.

Bath, P. A., & Morgan, K. (1999). Differential risk factor profiles for indoor and outdoor falls in older people living at home in Nottingham, UK. *European Journal of Epidemiology, 15*(1), 65-73.

Bergland, A., Jarnlo, G. B., & Laake, K. (2003). Predictors of falls in the elderly by location. *Aging Clinical and Experimental Research, 15*(1), 43-50.

Bergen, C; Stevens, M.R; Burns ER. (2016). Falls and fall injuries among adults aged >65 years – United States, 2014. *MMWR Mmorb Mortal Wkly Rep. 65(*37):993-998.

Berkman, L., Singer, B., & Manton, K. (1989). Black/white differences in health status and mortality among the elderly. *Demography, 26*(4), 661-678.

Billick, S. B., Siedenburg, E., Burgert, W., & Bruni-Solhkhah, S. M. (2001). Validation of the Mental Alternation Test with the Mini-Mental State Examination in geriatric psychiatric inpatients and normal controls. *Comprehensive psychiatry, 42*(3), 202-205.

Cerin, E., Saelens, B. E., Sallis, J. F., & Frank, L. D. (2006). Neighborhood Environment Walkability Scale: validity and development of a short form. *Med Sci Sports Exerc, 38*(9), 1682-1691.

Chippendale, T., & Boltz, M. (2014). The Neighborhood Environment: Perceived Fall Risk, Resources, and Strategies for Fall Prevention. *The Gerontologist*, gnu019.

Erosheva, E. A. (2005). Comparing latent structures of the grade of membership, Rasch, and latent class models. *Psychometrika, 70*(4), 619-628.

Erosheva, E. A. (2006). Latent class representation of the grade of membership model. *Seatle: University of Washington*.

Folstein, M. F., Folstein, S. E., & McHugh, P. R. (1975). “Mini-mental state”: a practical method for grading the cognitive state of patients for the clinician. *Journal of psychiatric research, 12*(3), 189-198.

Gallagher, N. A., Clarke, P. J., Ronis, D. L., Cherry, C. L., Nyquist, L., & Gretebeck, K. A. (2012). Influences on neighborhood walking in older adults. *Research in gerontological nursing, 5*(4), 238.

Gu, D., & Zeng, Y. (2012). Healthiness of survival and quality of death among oldest old in China using fuzzy sets. *Journal of Aging and Health, 24*(7), 1091-1130.

Guralnik, JM; Ferrucci, L; Pieper, CF e al. (2000). Lower extremity function and subsequent disability: consistency across studies, predictive models, and value of gain speed along compared with the short physical performance battery. *Journal of Gerontology: Medical sciences 55*(4):M221-M231

Guralnik, J. M., Ferrucci, L., Simonsick, E. M., Salive, M. E., & Wallace, R. B. (1995). Lower-extremity function in persons over the age of 70 years as a predictor of subsequent disability. *New England Journal of Medicine, 332*(9), 556-562.

Guralnik, J. M., & Simonsick, E. M. (1993). Physical disability in older Americans. *J Gerontol, 48 Spec No*, 3-10.

Guralnik, J. M., Simonsick, E. M., Ferrucci, L., Glynn, R. J., Berkman, L. F., Blazer, D. G., et al. (1994). A short physical performance battery assessing lower extremity function: association with self-reported disability and prediction of mortality and nursing home admission. *Journal of gerontology, 49*(2), M85-M94.

Kelsey, J. L., Procter-Gray, E., Hannan, M. T., & Li, W. (2012). Heterogeneity of falls among older adults: Implications for public health prevention. *American Journal of Public Health*(0), 1-8.

Li, W., Keegan, T. H. M., Sternfeld, B., Sidney, S., Quesenberry Jr, C. P., & Kelsey, J. L. (2006). Outdoor falls among middle-aged and older adults: A neglected public health problem. *American Journal of Public Health, 96*(7), 1192.

Li, W., Procter-Gray, E., Lipsitz, L. A., Leveille, S. G., Hackman, H., Biondolillo, M., et al. (2014). Utilitarian walking, neighborhood environment, and risk of outdoor falls among older adults. *American Journal of Public Health, 104*(9), e30-e37.

Lo, A.X., Rundle, A.G.;Buys, D; Dennedy R,E,; Sawyer, R,;Allman, R,MM.; and Brown C.J. (2016). Neighborhood disadvantage and life-space mobility are associated with incident falls in community-welling older adutls. *Journal of the American Geriatric Society 64*(11):2218-2218.

Maetzel, A., Johnson, S. H., Woodbury, M., & Bombardier, C. (2000). Use of grade membership analysis to profile the practice styles of individual physicians in the management of acute low back pain. *Journal of clinical epidemiology, 53*(2), 195-205.

Merom, D., Gebel, K., Fahey, P., Astell-Burt, T., Voukelatos, A., Rissel, C., et al. (2015). Neighborhood walkability, fear and risk of falling and response to walking promotion: The Easy Steps to Health 12-month randomized controlled trial. *Preventive Medicine Reports, 2*, 704-710.

Nagel, C. L., Carlson, N. E., Bosworth, M., & Michael, Y. L. (2008). The relation between neighborhood built environment and walking activity among older adults. *American Journal of Epidemiology, 168*(4), 461-468.

Nagi, S. Z. (1976). An epidemiology of disability among adults in the United States. *The Milbank Memorial Fund Quarterly. Health and Society*, 439-467.

Pomarol-Clotet, E., Salvador, R., Murray, G., Tandon, S., & McKenna, P. J. (2010). Are there valid subtypes of schizophrenia? A grade of membership analysis. *Psychopathology, 43*(1), 53-62.

Reitan, R. M. (1958). Validity of the Trail Making Test as an indicator of organic brain damage. *Perceptual and motor skills, 8*(3), 271-276.

Rosow, I., & Breslau, N. (1966). A Guttman health scale for the aged. *Journal of gerontology*(21), 556-559.

Rubenstein, L. Z., & Josephson, K. R. (2006). Falls and their prevention in elderly people: what does the evidence show? *Medical Clinics of North America, 90*(5), 807-824.

Saelens, B. E., Sallis, J. F., Black, J. B., & Chen, D. (2003). Neighborhood-based differences in physical activity: An environment scale evaluation. *American Journal of Public Health, 93*(9), 1552-1558.

Satariano, W. A., Ivey, S. L., Kurtovich, E., Kealey, M., Hubbard, A. E., Bayles, C. M., et al. (2010). Lower-body function, neighborhoods, and walking in an older population. *American Journal of Preventive Medicine, 38*(4), 419-428.

Satariano, W.A., Scharlach A.E., & Lindeman D. (2014). Aging, place, and technology: Toward improving access and wellnes in older populations. *Journal of Aging and Health*, 26, 1373-1389.

Speechley, M., & Tinetti, M. (1991). Falls and injuries in frail and vigorous community elderly persons. *Journal of the American Geriatrics Society, 39*(1), 46-52.

Stalvey B.T., Owsley C., Sloane, M., Ball, Karlene. (1999). Life space questionnaire: A measure of the extent of mobility of older adults. *Journal of Applied Gerontology,* 18(4), 460-478.

VanSwearingen, J. M., & Brach, J. S. (2001). Making geriatric assessment work: selecting useful measures. *Physical Therapy, 81*(6), 1233-1252.
